# Key Outcomes for Evaluating Hand and Wrist Scars: A Nationwide Survey of Clinicians in Saudi Arabia

**DOI:** 10.3390/medicina62030459

**Published:** 2026-02-28

**Authors:** Hadeel R. Bakhsh, Raghad W. Alotaibi, Monira I. Aldhahi, Donna L. Kennedy

**Affiliations:** 1 Department of Rehabilitation Sciences, College of Health and Rehabilitation Sciences, Princess Nourah bint Abdulrahman University, Riyadh 11564, Saudi Arabia; hrbakhsh@pnu.edu.sa (H.R.B.); mialdhahi@pnu.edu.sa (M.I.A.); 2 Therapies Department, Imperial College Healthcare NHS Trust, London W2 1NY, UK; d.kennedy@imperial.ac.uk; 3 Human Performance Group, Department of Surgery & Cancer, Imperial College London, London SW7 2AZ, UK

**Keywords:** hand therapy, scar management, outcome measures, rehabilitation

## Abstract

*Background and Objectives:* Hand and wrist scars alter physical appearance and can result in functional impairments and psychosocial difficulties. Although these effects are clinically important, rehabilitation services in Saudi Arabia lack consistent and standardised scar assessment protocols. The limited use of validated outcome measures hinders both clinical practice and research. Standardised scar assessment is essential for evidence synthesis, developing new scar care interventions and promoting best outcomes. We aim to investigate healthcare professionals’ perspectives on key scar outcome domains for evaluating hand and wrist scars and identify gaps in current practice and training needs to support the development of evidence-based guidelines. The study design is a cross-sectional descriptive study. *Materials and Methods:* The Saudi Commission for Health Specialties distributed a survey to 5000 randomly selected licensed healthcare professionals. The adapted questionnaire obtained sociodemographic data, professional experience, and ratings of scar outcome domains using a five-point Likert scale. Descriptive statistics were used for the analyses. *Results:* The analysis included 74 completed responses (response rate, 41.5%). Nurses (32.4%) and occupational therapists (29.7%) represented the largest groups. Only 37.8% of the participants reported receiving specialised training in scar assessment. Furthermore, the use of outcome measures remained limited, with 41.3% utilising clinician-reported outcome measures (CROMs) and 54.05% using patient-reported outcome measures (PROMs). The Vancouver Scar Scale and Patient and Observer Scar Assessment Scale were the most frequently used assessment tools. Clinicians primarily evaluated physical symptoms, including hypersensitivity (69.8%) and pain (67.6%), as well as scar characteristics such as colour (62.2%), adhesion (65.8%), and thickness (64.9%). Psychological factors were also considered important, particularly self-confidence (59.5%), acceptance of the scar (60.3%), and satisfaction with the scar (60.8%). *Conclusions:* Healthcare professionals in Saudi Arabia recognise the complex effects of hand and wrist scarring; however, they show limited integration of validated assessment tools, especially patient-reported outcome measures, in clinical practice. This gap suggests the need for targeted training, interdisciplinary educational initiatives, and efforts to strengthen standardised approaches to scar assessment. Exploring the development of future national guidance and engaging in international efforts to develop a core outcome measurement set may support evidence-based evaluation and improved long-term patient outcomes.

## 1. Introduction

Pathological scar formation is a significant negative outcome of injuries or surgeries. Scarring can be burdensome, leading to unpleasant or painful symptoms that hinder daily activities and social engagement [1]. Annually, it is estimated that more than 100 million individuals develop scars, mainly due to surgical interventions [1]. Currently, the global market for scar treatment was valued at $20 billion in 2020 and is expected to surpass $60 billion over the next decade [2]. These global economic trends highlight the substantial healthcare and financial burden associated with suboptimal scar management.

In Saudi Arabia, the impact of hand and wrist injuries is notably high, with the country recording the highest age-standardized incidence rate of upper extremity fractures in the Middle East and North Africa region in 2019, at 2296.93 per 100,000 individuals, significantly above regional averages [3]. Among these upper extremity fractures, wrist and distal hand fractures constitute a major injury category, with falls being the primary cause, resulting in 658,202 fracture cases in 2019 [3].

In the field of upper limb rehabilitation, managing scars on the hand and upper limb following traumatic injury or surgery is routine practice [4], and it is widely recognised as a vital aspect of this discipline [5,6]. According to the 2019 practice analysis survey conducted by the Hand Therapy Certification Committee, scar management is identified as a key skill necessary for improving patient outcomes in hand therapy [7].

Recently, the British Society for Surgery of the Hand—James Lind Alliance Priority Setting Partnership identified research to improve scarring following hand surgery or trauma as a top ten priority [8]. However, developing and testing new scar interventions requires a standardised approach to evaluation. In contrast, the current literature shows substantial heterogeneity in outcome reporting, limited use of validated tools, and a lack of agreed-upon terminology [5,9,10,11,12]. A recent state-of-the-art review led by Kennedy et al. revealed that only 25% of clinical hand and wrist studies utilised standardised scar outcome measures, and only 7% included patient-reported outcomes (PROMs) [11]. Most studies focused on scar symptoms but lacked consistent definitions or robust measurement methods.

Despite the significant impact of scarring following hand injuries or surgeries, there is an absence of standardised, evidence-based protocols for assessing and managing scars in Saudi rehabilitation services. This gap is further reflected in the broader literature, which demonstrates substantial inconsistency in outcome reporting and limited use of validated tools. Therefore, there is an urgent need for enhanced specialty-based rehabilitation services, including the development of national guidelines to support consistent, evidence-based scar evaluation [3].

This study aimed to investigate the current clinical practice of Saudi health professionals in evaluating scars following surgery and injuries to the hand and upper limb. Obtaining insights into clinicians’ skill development and scar evaluation methods supports the identification of training needs and informs the clinical research agenda.

## 2. Materials and Methods

### 2.1. Study Design

This cross-sectional descriptive study used a survey design to reach the greatest number of health care providers providing hand scar care across geographical regions in Saudi Arabia. A random sample of 5000 health professionals was invited through the Saudi Council for Health Specialties (SCFHS), a professional organisation responsible for regulating and accrediting healthcare-related practices and practitioners at all levels within the Kingdom. The invitation targeted professionals working in specialties that commonly manage hand and wrist injuries or scars. Participation was voluntary. The “Checklist for Reporting Results of Internet E-Surveys” (CHERRIES) guideline was used to inform both the design and reporting of this study (Appendix A) [2].

### 2.2. Ethical Consideration

Approval from the local Institutional Review Board for Ethics was obtained from Princess Nourah Bint Abdulrahman University (IRB Registration Number with KACST, KSA: HAP-01-R-059, PNU IRB Log Number: 24-0936). This study was conducted in accordance with the Declaration of Helsinki.

### 2.3. Survey Instrument

A self-administered electronic questionnaire was adapted from a previous study conducted by Kennedy et al. [3]. Permission was obtained from the first author [H.R.B] to use the survey (personal communication).

The survey consisted of two sections (Supplementary Materials File S1). The first section queried the sociodemographic information of the participants (i.e., profession, city of practice, years of experience, type of practice setting, self-reported level of expertise in scar evaluation, clinical populations treated or assessed, and whether the survey participant was a clinician, academic, or clinical academic). The second section consisted of seven questions asking participants to rate the importance of assessing various scar outcome domains, such as the use of standardised patient- or clinician-reported outcome measures, physical symptoms, scar characteristics, functional impairment, hand function, and psychological impact. For each domain, and again at the end of the survey, participants were invited to provide additional input through open-ended responses. Domain-specific questions were phrased, for example, as: “Do you think it is important to evaluate any of the following physical characteristics of a scar?” Responses were rated on a 5-point Likert scale: definitely not (1), probably not (2), neutral/unsure (3), probably yes (4), and definitely yes (5).

The questionnaire was reviewed by experienced clinicians and researchers in the field of hand and upper limb scar assessment to ensure clarity and clinical relevance. Feedback was used to refine item wording prior to distribution.

### 2.4. Participants and Procedure

The survey was approved for distribution by the SCFHS to licenced targeted health professions and was considered a closed survey. Participants were invited through a generic email via the SCFHS mailing list to a random sample of 5000 registrants. The SCFHS database team conducted the random selection process using their internal automated sampling system. The research team did not have access to the registry, and randomisation was performed independently by SCFHS staff to ensure unbiased selection. To be eligible, professionals with relevant backgrounds working in any city in Saudi Arabia were required to be licenced and registered to practice in Saudi Arabia through the SCFHS. The invitation targeted the following professions: plastic surgeons, orthopaedic surgeons, dermatologists, occupational therapists, physiotherapists, nurses, and other related disciplines. Participants were excluded if they were students or health professionals who were not currently working in Saudi Arabia. The researchers were blinded to the identities of the potential participants.

The survey was disseminated through the SCHFS via the online survey program Qualtrics XM (Seattle, WA, USA) which is primarily managed by the organisation. Participation was voluntary, and the participants were not reimbursed. The Survey was open from February to June 2025. A first reminder was sent 4 weeks from the start of the survey and a second reminder 4 weeks prior to the closure of the survey. Participants provided consent by agreeing to the opening questions. To ensure the confidentiality and anonymity of the participants, no identifying information was collected. The investigators did not have access to participant data (i.e., names, contact details, and licence numbers). The investigators were only able to access the results of the survey provided by the SCFHS in Excel format.

### 2.5. Data Analysis

A descriptive statistical analysis was conducted to summarise the participants’ responses. Data from Likert-scale items were analysed using frequencies and percentages for each response category to represent participants’ ratings across all scar outcome domains. This approach allowed for a clear understanding of how the respondents prioritised the importance of various scar outcome domains. The distribution of responses was examined to identify areas of strong agreement, uncertainty, or divergence among the participants, providing insights into the consensus levels across different aspects of scar assessment. Only fully completed questionnaires were included in the analysis. All partially completed responses and entries with missing or inconsistent data were excluded during data cleaning. Qualtrics’ built-in settings were used to minimise duplicate submissions. No imputation procedures were applied. All analyses were performed using Microsoft Excel (version 16.0; Microsoft Corporation, Redmond, WA, USA).

## 3. Results

### 3.1. Participant Sociodemographic

A total of 185 clinicians responded to the survey, and six declined to participate. Of the 178 clinicians who agreed to participate, only 74 submitted a completed response; 111 questionnaires were incomplete and thus excluded from the analysis. The remaining seventy-four survey responses were included in the data analysis. This represents a completion rate of 41.5% among those who initiated the survey and an overall response rate of 1.48% (74 of 5000 invitations) distributed by the SCFHS. A summary of the participants’ sociodemographic characteristics is presented in Table 1. The frequency of scar care varied widely among participants, and only 37.8% of the participants reported receiving specialised training in scar assessment or management (Table 2).

### 3.2. Patient-Reported Outcome Measures (PROMs)

Fifty-four percent of participants reported using PROMs, while 46% had never heard of these measures. The Patient and Observer Scar Assessment Scale (POSAS) [4] was the most frequently cited, used by 40% of all participants (Table 2).

### 3.3. Clinician-Reported Outcome Measures (CROMs)

The use of standardised PROMs showed higher adoption than CROMs, with 54% of participants reporting the use of PROMs compared to 41.89% for CROMs. However, 33.78% reported that they had never heard of the measures listed, and 24.32% were aware but did not use any CROMs. Among participants who reported using CROMs, the Vancouver Scar Scale (VSS) [5] was the most frequently employed clinician-reported measure, used by 67.74% of this subgroup. Other measures, such as the Stony Brook Scar Evaluation Scale [6] and the Matching Assessment of Scars and Photographs (MAPS)/Manchester Scar Scale [7] had much lower usage (Table 2).

### 3.4. Physical Symptoms and Characteristics

**Figure 1:**
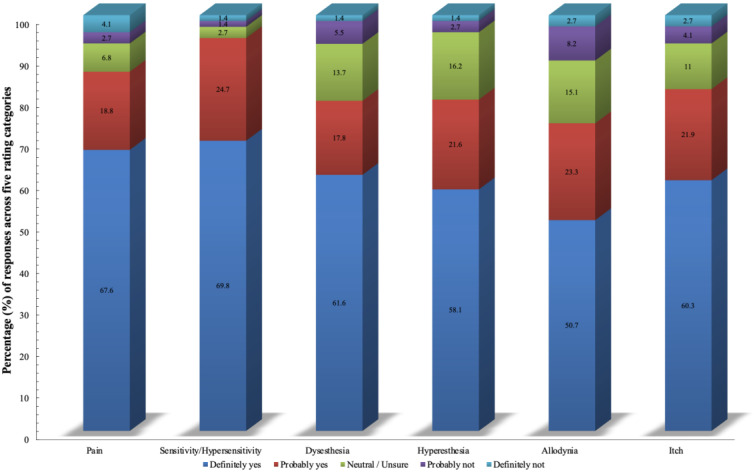
Importance of assessing physical symptoms in hand scar evaluation (N = 74).

Figure 1 demonstrates respondent priorities in evaluating scar physical symptoms. Sensitivity/hypersensitivity was rated the highest, with 94.6% of participants endorsing it as important, followed by pain (86.5%), while allodynia showed the lowest (but still substantial) importance rating. The data show a strong consensus that physical symptoms should be assessed, with all symptoms receiving majority support.

**Figure 2:**
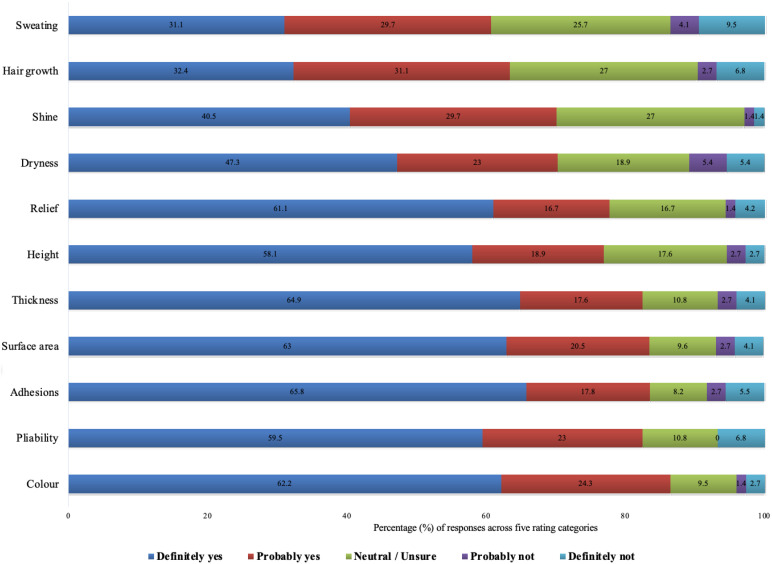
Clinician-rated importance of assessing physical scar characteristics (N = 74). The stacked bars show the percentage of responses across the five rating categories for each feature.

For physical characteristics, adhesion, surface area, and colour were the most critical features to be assessed, with 83.6%, 83.5%, and 86.5% endorsement, respectively (Figure 2). Thickness and pliability followed closely at 82.5% each. Features such as hair growth (59.4%) and sweating (56.8%) garnered lower but still majority support, reflecting a more variable emphasis on these surface phenomena. Additional symptoms mentioned by participants as free text include tenderness, functional limitations, cracking/skin breakdown, appearance, and complicated scar development.

### 3.5. Physical Impairment Measure

**Figure 3:**
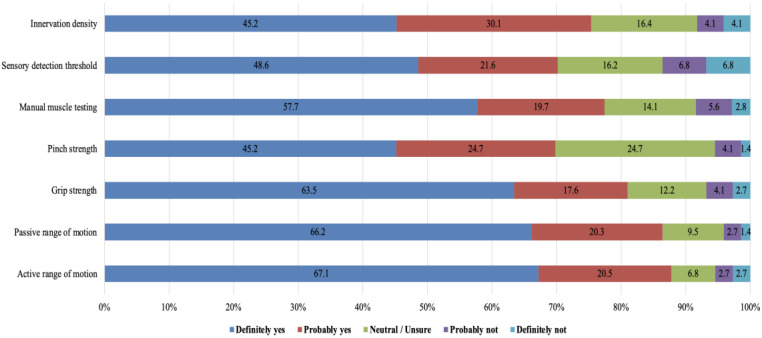
Importance of key impairment measures in hand scar assessment (N = 74). The stacked bars represent the proportion of clinicians rating each functional test importance as part of their routine scar evaluation practice.

When evaluating impairment measures (Figure 3), active and passive range of motion led with 87.6% and 86.5% high-importance endorsement, respectively. Grip strength (81.1%) and pinch strength (77.4%) were also considered critical. Measures such as sensory detection threshold (75.3%), innervation density (74.3%), manual muscle testing (70.2%), and dynamometry (69.9%), although valued by most respondents, showed larger neutral shares, indicating some variability in their perceived necessity.

### 3.6. Psychological Impact

**Figure 4:**
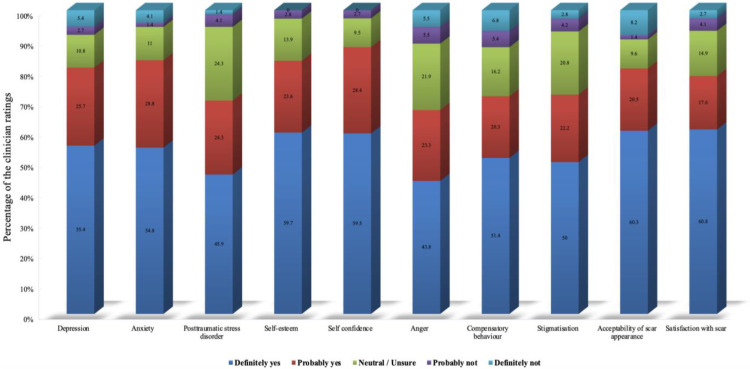
Importance of assessing the mental health impacts of hand scars (N = 74). The stacked bars show clinician ratings across ten psychosocial domains.

Self-perception and affective domains emerged as the most critical mental health impacts to assess in patients with scarring (Figure 4). Satisfaction with one’s scar (4%) and acceptability of appearance (8%) received the highest endorsement, underscoring clinicians’ prioritisation of patients’ overall acceptance and comfort with their appearance. Self-confidence (9%) and self-esteem (3%) followed closely, highlighting the psychosocial burden associated with visible scarring. In contrast, anger showed somewhat lower but still majority support.

## 4. Discussion

This cross-sectional study investigated the domains of scar assessment and treatment considered important by clinicians experienced in evaluating hand and wrist scars in adults. This study provides significant insights into the current clinical practice and perspectives on scar assessment among healthcare professionals in Saudi Arabia, particularly concerning hand and wrist injuries. Despite the substantial national burden of upper extremity fractures and the well-documented psychosocial and functional consequences of scarring, our findings highlight critical gaps in routine assessment practices and training across various healthcare professional groups in Saudi Arabia.

At the outset, the limited survey response rate may suggest that scarring receives scant attention regionally and that scar assessment and care are not a clinical priority, despite the extensive burden of scarring. Congruently, this survey highlights that clinicians receive limited training in scar assessment and management, with only 37.8% of participants reporting receiving such training. This aligns with previous research indicating a general lack of expertise in burn management among Saudi healthcare professionals, suggesting a broader issue in specialized scar care training within the region. This deficiency is particularly concerning given that up to 40% of patients with burn injuries will develop hand scar contractures, significantly compromising function and quality of life. Exploring how education in scar evaluation and management might be prioritised in cross-professional clinical training is an important area for future development.

While there is good evidence that holistic scar care requires capturing the patients’ experience of scarring [6,11], notably, survey participants reported limited knowledge and low adoption of standardized patient-reported outcome measures (PROMs). Nearly half (45.9%) of participants had limited exposure to or reported no awareness of PROMs. Among clinicians with awareness of PROMs, most did not integrate these measures into routine clinical practice. This finding suggests that patients’ subjective experience of scarring is underestimated, rather than central to care. Thus, the lack of PROM adoption identified in our study echoes Kennedy et al. (2023), who found that only 7% of clinical studies on the hand and wrist incorporated hand scar PROMs [13,14].

Survey results indicate that 54% of participants regularly use PROMs in clinical practice. This suggests an awareness that a comprehensive, patient-centered scar evaluation requires assessment of subjective domains alongside objective clinical measurements and is a promising start for uptake. However, given the descriptive nature of our study and low overall response rate, these findings should be interpreted cautiously. Our findings may reflect the perspectives of clinicians already engaging in scar care, rather than those of the wider workforce [15]. From an implementation science perspective, uptake is likely at its earliest stage, wherein the healthcare community becomes more aware of the advantages these tools provide for patient care.

Survey findings suggest participants have a substantial knowledge gap regarding CROMs. Numerous tools have been developed for clinician-completed scar assessment; however, as Carrière et al. (2019) (2023) pointed out, scar evaluation is not standardised across various fields of medicine [4,16]. Furthermore, heterogeneity in instrument design and content preclude comparability and synthesis, impeding advances in scar care [4,16]. In this survey, 68% of participants reported using the VSS and 40% the POSAS, aligning with international trends in scar assessment [16,17,18]. However, the utilisation of other standardised CROMs, such as MAPS and Stony Brook, was notably infrequent. This limited diversity in tool application may stem from a lack of training, limited exposure to evidence-based tools, or insufficient institutional support. These findings parallel those of Scott et al. (2024), who pointed out a major gap in clinicians’ education in assessing scars, poor awareness, insufficient inter-professional education, and the absence of local guidelines as key barriers to standardised practice [19]. Interestingly, one participant noted in the “other” section: “I didn’t know that there are other things than VSS”—highlighting that VSS dominates awareness in this field.

Furthermore, investigators reinforce the need for alignment between standardisation and patient-centredness. Price et al. (2021) [11] demonstrated that patients value subjective interpretation and continuity of care over purely technical assessments, though validated tools like the POSAS can foster reflection and communication. Moreover, practical barriers, such as limited training and poor awareness of outcome tools, as noted by Scott et al. (2024) [19], challenge the uptake of CROMs in clinical environments.

Beyond outcome tool usage, the survey revealed that clinicians recognise the multidimensional nature of scars, particularly in assessing physical characteristics, symptoms, functional impairments, and psychological impacts. This comprehensive approach aligns with the established understanding that scar assessment should encompass cosmetic appearance, physical symptoms, functional loss, and psychological or social problems to truly capture patient well-being [20]. Clinicians reported prioritising the evaluation of physical symptoms, strongly agreeing that scar colour, pliability, adhesion, size, and thickness are important characteristics. In contrast, characteristics associated with surface phenomena, such as dryness, hair growth, and sweating, generated a varied range of responses. This divergence may reflect a perceived lesser clinical significance for these attributes despite their potential impact on appearance and comfort, especially in specific environmental contexts, such as warmer climates.

The physical characteristics of the scar, including hypersensitivity and pain, were consistently rated as high-priority evaluation domains. This aligns with the existing literature highlighting the functional limitations and discomfort caused by these features, as they can impair mobility and limit quality of life [14,21]. Likewise, strong endorsement for evaluating impairment measures including active and passive range of motion, grip and pinch strength, and sensory thresholds illustrates clinicians’ understanding of the close link between scar characteristics and overall hand function.

Interestingly, there was notable support for evaluating psychological impacts, with self-confidence, satisfaction with appearance, and acceptability of scars rated as the most critical. This holistic perspective resonates with research emphasising that scars negatively impact mental health, self-esteem, body image, and can lead to social impairment, depression, and anxiety [22,23,24]. Thus, Kennedy et al.’s (2023) [3] conclusion is that scar assessment should balance standardised metrics with subjective, patient-derived concerns. The alignment between clinician responses in this study and the domains identified in Kennedy’s survey of international clinicians is promising. This agreement suggests a readiness among Saudi clinicians to adopt more comprehensive, patient-centred practices, although these interpretations should be approached cautiously given the potential self-selection bias affecting our sample.

Curiously, this study revealed inconsistencies in the prioritisation of scar evaluation domains. For example, surface characteristics like sweating or hair growth were perceived as less important, despite literature suggesting that these features may significantly affect appearance and comfort, particularly in hotter climates like Saudi Arabia. Similarly, while satisfaction with scars was rated highly, psychological domains such as anger and post-traumatic stress received less consistent support. This variability may reflect discomfort or lack of training in addressing mental health in clinical rehabilitation settings, a challenge also identified by Deflorin et al. (2020) and Price et al. (2021) [11,21]. Their survey of hand therapists and surgeons indicated that, despite the availability of these tools, their utilisation remains inconsistent. This variation can be largely explained by systemic barriers and education-related issues. These pragmatic issues may represent a barrier to the future development and uptake of a Core Outcome Set for hand scars [3,13,25].

This study has some limitations that warrant consideration. The low response rate introduces the risk of response bias; it is likely that those with an existing interest in scars were more inclined to participate. The predominance of early- to mid-career professionals may also skew the results toward less experienced perspectives, especially given the limited training in scar assessment. Moreover, the use of descriptive statistics limits inferences about associations between profession, training, and outcome prioritisation; future studies should explore these relationships more robustly using inferential analysis. Although this study employed a descriptive cross-sectional survey design that captured a comprehensive perspective of current practices among healthcare professionals regarding hand and wrist scar assessment across Saudi Arabia, determining causality is naturally limited by the study’s design. Furthermore, the results of a study that mostly included respondents from the capital region might not be generalisable to other healthcare systems or cultural contexts. Future qualitative studies should broaden the geographic scope to include a range of populations and healthcare environments. Additionally, the very low overall response rate (1.48%) limits representativeness and generalisability, as non-respondents may differ substantially from those who completed the survey.

Despite these limitations, this study makes a timely and valuable contribution to clinical rehabilitation in Saudi Arabia. It offers the first empirical insight into local perspectives on scar assessment post-hand and wrist injury and highlights a clear appetite for improvement. However, the interpretations of national-level needs should be framed cautiously. Rather than providing definitive evidence for wide-scale implementation, these findings may help inform future planning and discussions around potential capacity-building initiatives, such as workshops, interdisciplinary training, or the integration of validated scar outcome measures into electronic medical records. Moreover, while the data suggest areas where guidance could be developed, they should not be interpreted as direct evidence for immediate national protocol implementation. Standardisation of evaluation remains important for evidence synthesis and improving clinical practice, but such recommendations represent future implications rather than conclusions directly drawn from this sample.

## 5. Conclusions

While healthcare professionals in Saudi Arabia recognise the complex nature of hand and wrist scarring, including its physical, functional, and psychological dimensions, their clinical evaluation practices remain variable, reflecting the current absence of internationally agreed standards. Limited awareness, training, and system-level support may contribute to the reduced adoption of validated assessment tools. Given the relatively small sample size and low response rate, the findings of the present study should be interpreted as exploratory and descriptive, and the proposed implications should be viewed as potential future directions rather than conclusions directly supported by causal evidence. Moving forward, healthcare leaders and educators could consider prioritising scar assessment as a core component of upper limb rehabilitation. Exploring the future development of a standardised yet flexible scar management framework, informed by ongoing international efforts to establish core outcome sets and tailored to local realities, may help support improved functional recovery, aesthetic outcomes, and quality of life for patients across Saudi Arabia.

Overall, the purpose of this study was to provide an initial overview of current clinical practices and training needs related to hand and wrist scar assessment in Saudi Arabia, which may guide future research, guideline development, and educational initiatives. It may also be valuable to further develop the scar management expertise of clinicians. Gaining insight into healthcare professionals’ present practices, treatment inclinations, and requirements for clinical development can facilitate the identification of training needs and provide direction for the future creation of evidence-based guidelines and educational initiatives.

## Figures and Tables

**Table 1:** Sociodemographic Characteristics of Participants (N = 74).

Variables		N (%)
Profession	Nurse	24 (32.4%)
	Occupational Therapist	22 (29.7%)
	Other medical specialisations	13 (17.6%)
	Hand surgeon (Plastics)	8 (10.8%)
	Physiotherapist	5 (6.8%)
	Dermatologist	2 (2.7%)
Healthcare Setting	Public Hospital	49 (66.21%)
	Private Practice:	11 (14.9%)
	Rehabilitation Centre	5 (6.75%)
	Other	4 (5.41%)
	Academic/Research Institution	3 (4.05%)
	Military Hospital	1 (1.35%)
	Community Health Centre	1 (1.35%)
Level of Experience	Intermediate	35 (47.3%)
	Novice	21 (28.3%)
	Expert	18 (24.3%)
Area of Practice	Clinician	60 (81.08%)
	Clinical Academic	11 (14.86%)
	Academic/Researcher	3 (4.05%)
Years of Experience	Less than 5 years	29 (39.2%)
	5–10 years	23 (31.1%)
	11–15 years	10 (13.5%)
	More than 20 years	7 (9.5%)
	16–20 years	5 (6.8%)
Region	Central (i.e., Riyadh)	51 (68.91%)
	Western	13 (17.56%)
	Southern	4 (5.40%)
	Eastern	3 (4.05%)
	Northen	3 (4.05%)

**Table 2:** Clinical Training and Practice of Participants (N = 74).

Variables		N (%)
Specialised training in scar assessment and management	No	46 (62.2%)
	Yes	28 (37.8%)
Frequency of clinical encounters involving hand and wrist scars	Occasionally (a few times a month)	35 (47.3%)
	Rarely (less than once a month)	21 (28.3%)
	Regularly (weekly)	18 (24.3%)
Most commonly evaluated patient populations	A combination of the above	54 (73.0%)
	Burns	7 (9.5%)
	Elective hand surgery	5 (6.8%)
	Orthopaedic trauma (i.e., open reduction and internal fixation)	3 (4.1%)
	Trauma (i.e., flexor tendon repair)	3 (4.1%)
	Complex trauma (i.e., traumatic, open multi-structure injuries)	2 (2.7%)
Patient-Reported Outcome Measure (PROMs) Usage	Uses PROMs	40 (54.05%)
	Never heard of these measures	34(45.9%)
Reported PROMs Used	Patient and Observer Scar Assessment Scale (POSAS)	16 (40%)
	Patient Scar Assessment Questionnaire (PSAQ)	8 (20%)
	Patient-Reported Impact of Scars Measure (PRISM)	7 (17.5%)
	Scar Quality of Life Questionnaire (SCAR-Q)	7 (17.5%)
	University of North Carolina 4P Scar Scale (UNC4P)	2 (5%)
Clinician-Reported Outcome Measures (CROMs) usage	Uses clinician measures	31 (41.89%)
	Never heard of these measures	25 (33.78%)
	Do not use any clinician measures	18 (24.32%)
Types of CROMs used	Vancouver Scar Scale (VSS)	21 (67.74%)
	Stony Brook Scar Evaluation Scale (SBSES)	4 (12.90%)
	Matching Assessment of Scars and Photographs (MAPS)	2 (6.45%)
	Manchester Scar Scale (MSS)	3(9.67%)
	Silverberg Scar Mobility Rating Scale	1 (3.22%)

## Data Availability

The data presented in this study is available on request from the corresponding author. The data are not publicly available due to restrictions, e.g., their containing information that could compromise the privacy of research participants.

## Supplementary Materials

The following supporting information can be downloaded at: https://www.mdpi.com/article/10.3390/medicina62030459/s1. File S1. Scar Assessment Survey.

## Appendix A

While most items on the checklist are self-explanatory, a few comments about the “response rate” are in order. In traditional surveys investigators usually report a response rate (number of people presented with a questionnaire divided by the number of people who completed the questionnaire) to allow some estimation of the degree of representativeness and bias. Surveys with response rates lower than 70% or so (an arbitrary cut-off point!) are usually viewed with skepticism.

In online surveys, there is no single response rate. Rather, there are multiple potential methods for calculating a response rate, depending on what is chosen as the numerator and denominator.

**Table A1:** Checklist for Reporting Results of Internet E-Surveys (CHERRIES).

Item Category	Checklist Item	
**Design**	A cross-sectional descriptive study on random sample of 5000 health professionals in Saudi Arabia	
**IRB (Institutional Review Board) approval and informed consent process**	The local Institutional Review Board for Ethics was obtained from Princess Nourah Bint Abdulrahman University (IRB Registration Number with KACST, KSA: HAP-01-R-059, PNU IRB Log Number: 24-0936). Participants provided consent by agreeing to the opening question Participants were invited to complete the survey through a generic email via the SCFHS mailing list to a random sample of 5000 registrants. The researchers were blind to potential participants’ identities. No identifying information was collected in addition to the fact that the authors did not have access to the list of participants (i.e., names, contact details, licence number). Authors were only able to access the results of the survey.	
**Development and pre-testing**	A self-administered electronic questionnaire was adapted from a previous study done by Kennedy et al. [3]. A permission was obtained by the first author [H.R.B] to use the survey (Personal communication). Upon obtaining permission for the use of the questionnaire from the authors, face validation was carried out.	
**Recruitment process and description of the sample having access to the questionnaire**	The survey was approved to be distributed by SCFHS to licensed targeted health professions and was considered a closed survey. Participants were invited to complete the survey through a generic email via the SCFHS mailing list to a random sample of 5000 registrants.	
**Survey administration**	The survey was disseminated through SCHFS via online survey program Qualtrics XM (Seattle, WA, USA) which is primarily managed by the organisation, and participation was voluntarily. The Survey was open from February until June 2025; first reminder was sent 4 weeks from the start of the survey and the second reminder was 4 weeks prior to closure of the survey.	
	No incentives were given for participation No item randomisation No adaptive questioning No Completeness check Yes, there was a review step	
	Number of Items	The questionnaire consisted of 16 main items across two sections. Section 1 included 9 items (items 1–7 fully on page 1, and the remaining answers to item 7 plus items 8–9 on page 2). Section 2 included 7 items (4 table-type questions and 3 multiple-choice questions), starting mid-page 2 (items 1–3), continuing with items 4–6 partially on page 2 and partially on page 3, and concluding with item 7 on page 3. This distribution resulted in an average of approximately 5.33 items per page (16 items ÷ 3 pages).
	Number of screens (pages)	The survey was distributed across 3 pages. Page 1 contained items 1–7 (Section 1). Page 2 contained the remaining answers to item 7, items 8–9 (Section 1), and items 1–4 (Section 2). Page 3 contained the remaining answers to item 6 and item 7 from Section 2.
**Response rates**	based on IP addresses	
	View rate (Ratio of unique survey visitors/unique site visitors)	Not applicable. Qualtrics does provide data on the number of site visitors. Only number of those who started the survey was available.
	Participation rate (Ratio of unique visitors who agreed to participate/unique first survey page visitors)	96.2%. Out of 185 individuals who viewed the first page of the survey, 178 agreed to participate, while 6 declined and 2 exited before providing consent.
	Completion rate (Ratio of users who finished the survey/users who agreed to participate)	41.5%. Of the 178 participants who agreed to take part, 74 reached the final page of the questionnaire and submitted their responses. Completion here refers to reaching and submitting the final page, not necessarily answering every question in full.
**Preventing multiple entries from the same individual**	No Cookies used and no other log file analysis IP check was used to identify duplicates	
**Analysis**	Only complete responses were analysed and no Statistical correction	
	Questionnaires submitted with an atypical timestamp	Some investigators may measure the time people need to fill in a questionnaire and exclude questionnaires that were submitted too soon. Specify the timeframe that was used as a cut-off point and describe how this point was determined.
