# Supplementary material for: Key Outcomes for Evaluating Hand and Wrist Scars: A Nationwide Survey of Clinicians in Saudi Arabia
Source: Medicina (Kaunas). 2026 Feb 28;62(3):459. doi: 10.3390/medicina62030459 (PMC13027436; doi:10.3390/medicina62030459)
Supplement: Supplementary file 1 [file medicina-62-00459-s001.zip › medicina-4144428-supplementary.pdf]

SUPPLEMENTARY MATERIALS 1

Key Outcomes for Evaluating Hand and Wrist Scars: A Nationwide Survey of Clinicians in Saudi Arabia

Hadeel R, Bakhsh[1], Raghad W. Alotaibi[1] ***,** Monira I. Aldhahi[1] , Donna L Kennedy[2,3]

[1]Department of Rehabilitation Sciences, College of Health and Rehabilitation Sciences, Princess Nourah bint Abdulrahman University, Riyadh, Saudi Arabia.

[2]Therapies Department, Imperial College Healthcare NHS Trust, London, UK

[3]Human Performance Group, Department of Surgery & Cancer, Imperial College London, London, UK

*** Corresponding Author**

Raghad W. Alotaibi Raghad.w.alotibi@gmail.com

Scar Assessment and Management Survey

Section S1: Sociodemographic

1. **What is your profession?**
   - o Hand Therapist
   - o Occupational Therapist
   - o Physiotherapist
   - o Nurse
   - o Orthotist / Prosthetist
   - o Hand Surgeon (Plastics)
   - o Hand Surgeon (Orthopaedics)
   - o Dermatologist
   - o Other (please type here)
2. **How would you rate your level of experience / expertise in scar evaluation?**
   - o Novice
   - o Intermediate
   - o Expert
3. **Where do you practice, i.e., in what country? Please write below:**

   ____

4. **In what patient population do you most frequently evaluate hand and wrist scars?**
   - Burns
   - Elective hand surgery (i.e., planned surgery such as carpal tunnel release)
   - Orthopaedic trauma (i.e., open reduction and internal fixation of distal radius fracture)
   - Trauma (i.e., flexor tendon repair)
   - Complex trauma (i.e., traumatic, open multi-structure injuries)
   - A combination of the above
5. **Would you describe yourself as a:**
   - Clinician
   - Academic / Researcher
   - Clinical Academic

6. How many years have you been practicing in your profession?
- Less than 5 years
- 5-10 years
- 11-15 years
- 16-20 years
- More than 20 years

7. In which type of healthcare setting do you primarily work?
- Hospital
- Private Practice
- Rehabilitation Center
- Academic/Research Institution
- Community Health Center
- Other (please specify)

8. Have you received any specialized training in scar assessment or management?
- Yes
- No

9. How frequently do you encounter hand and wrist scars in your practice?
- Rarely (less than once a month)
- Occasionally (a few times a month)
- Regularly (weekly)
- Frequently (daily)

Section S2 Evaluation of hand and wrist scars

1. **Do you use any of the following standardised patient-reported outcome measures (PROMs) for the evaluation of hand and wrist scars? Please tick or write in all that apply.**

- o I do not use any PROMs for the evaluation of hand and wrist scars
- o Patient and Observer Scar Assessment Scale (POSAS) [Draaijers Let al 2004]
- o Patient Scar Assessment Questionnaire (PSAQ) [Durani Pet al 2009]
- o Patient-Reported Impact of Scars Measure (PRISM) [Brown, B et al 201O]
- o University of North Carolina Scar Scale (UNC4P) [Hultman C et al 2014]
- o SCAR-Q [Klassen, A et al 2018]
- o Other (please add name of PROM)

2. **Do you use any of the following standardised clinician-reported outcome measures for the evaluation of hand and wrist scars? Please tick or write in all that apply.**

- o I do not use any clinician completed standardised scar outcome measures
- o Vancouver Scar Scale (VSS) [Sullivan T. et al 1990]
- o Silverberg Scar Mobility Rating Scale [Silverberg R et al 1996]
- o Manchester Scar Scale (MSS) [Beausang E. et al 1998]
- o Matching Assessment of Scars and Photographs (MAPS) [Masters M et al 2005]
- o Stony Brook Scar Evaluation Scale (SBSES) [Singer et al 2007(
- o Other (please add name of scar outcome measure)

3. **Do you think it is important to evaluate any of the following scar physical symptoms?**

	Definitely not	Probably not	Neutral I Unsure	Probably yes	Definitely yes
Pain					
Sensitivity/ Hypersensitivity					
Dysesthesia					
Hyperesthesia					
Allodynia					
Itch					
Other (please write in)					

4. **Do you think it is important to evaluate any of the following physical characteristics of scar?**

	Definitely not	Probably not	Neutral I Unsure	Probably yes	Definitely yes
Pliability					
Adhesions					
Height					
Surface area					
Thickness					
Relief					
Hair growth					
Sweating					
Dryness					
Colour					
Shine					
Other (please write in)					

5. **Do you think it is important to evaluate any of the following impairment measures when assessing scars in the hand / wrist?**

	Definitely not	Probably not	Neutral / Unsure	Probably yes	Definitely yes
Active range of motion					
Passive range of motion					
Grip strength (Jamar					
dynamometry or other)					
Pinch strength					
Manual muscle testing					
Sensory detection threshold (i.e., Semmes-Weinstein monofilaments)					
Innervation density (i.e., -2 Point discrimination)					
Other (please write in)					

6. **When evaluating the functional impact of scars in the hand and wrist, do you use a standardised, patient-completed outcome measure (PROM)? Please tick or write in all that apply.**

- o I do not use a PROM to evaluate scar functional impact
- o Canadian Occupational Performance Measure (COPM) [Law et al 1990]
- o Boston Carpal Tunnel Questionnaire (BCTQ) [Levine D et al 1993]
- o Patient Evaluation Measure (PEM) [Macey et al 1995]
- o Disabilities of the Arm, Shoulder and Hand Outcome Measure (DASH) [Hudak PL et al 1996]
- o Patient Rated Wrist and Hand Evaluation (PRWHE) [MacDermid JC 1996]
- o Michigan Hand Questionnaire (MHQ) [Chung et al 1998]
- o Other (please write in)

7. **When evaluating the emotional or mental health impact of scars in the hand and wrist, do you think it is important to include any of the following factors or constructs? (Please tick or write in all that apply)**

	Definitely not	Probably not	Neutral I Unsure	Probably yes	Definitely yes
Depression					
Anxiety					
PosHraumatic stress disorder					
Self-esteem					
Self- confidence					
Anger					
Compensatory behaviour (to hide or conceal scar)					
Stigmatisation					
Acceptability of scar appearance					
Satisfaction with scar					
Other (please write in)					

Are there other domains or measures that should be included in the evaluation of scars in the hand and wrist? Please write in below (text):

____

____

____

Thank you for taking the time to participate in our survey!
